# Quantification of cell-free DNA for evaluating genotoxic damage from occupational exposure to car paints

**DOI:** 10.1186/s12995-016-0123-8

**Published:** 2016-07-15

**Authors:** Mónica Villalba-Campos, Sandra Rocío Ramírez-Clavijo, Magda Carolina Sánchez-Corredor, Milena Rondón-Lagos, Milcíades Ibáñez-Pinilla, Ruth Marien Palma, Marcela Eugenia Varona-Uribe, Lilian Chuaire-Noack

**Affiliations:** Facultad de Ciencias Naturales y Matemáticas, Universidad del Rosario, Carrera 26 63B-48, Bogotá, DC Colombia; Escuela de Medicina y Ciencias de la Salud, Universidad del Rosario, Bogotá, DC Colombia; Instituto Nacional de Salud, Bogotá, DC Colombia

**Keywords:** Car painters, Organic solvents, Occupational exposure, Genotoxicity, Cell-free DNA, Comet assay

## Abstract

**Background:**

For several years, cell-free DNA has been emerging as an important biomarker for non-invasive diagnostic in a wide range of clinical conditions and diseases. The limited information available on the genotoxic effects associated with occupational exposure to car paints, as well as the fact that up-to-date there are not reports about cell-free DNA measurements for assessing this condition, led us to evaluate the DNA damage caused by the occupational exposure to organic solvents contained in car paints, through the quantification of the cell-free DNA and the comet assay, in a sample of 33 individuals taken from 10 automobile paint shops located in Bogota DC, Colombia.

**Results:**

By applying the two methods, cell-free DNA and comet assay, we found a significant increase in the extent of DNA damage in the exposed individuals compared with the non-exposed ones within the control group.

**Conclusions:**

Our findings provide useful information about the cell-free DNA levels in this type of exposure and can be considered as a support tool that contributes to the diagnosis of genotoxic damage in individuals occupationally exposed to car paints.

**Electronic supplementary material:**

The online version of this article (doi:10.1186/s12995-016-0123-8) contains supplementary material, which is available to authorized users.

## Background

The aromatic hydrocarbons used as solvents and paint removers (BTX - benzene, toluene, xylene) have been included in the list of substances to which workers in the paint industry are exposed to, according to IARC 2010 report [1]. Although most of metabolic products of these solvents, such as the S-phenyl mercapturic and trans-trans-muconic acids derived from benzene, and the hippuric acid derived from toluene are eliminated through the urine, some intermediate metabolites can interact with DNA and alter its structure, which makes benzene causes certain types of hematological disorders and cancer [2, 3] and toluene exhibits its toxic properties mainly at neuronal, urinary and reproductive level [4], among others.

In the case of individuals occupationally exposed to car paints, an increase in oxidative damage has been demonstrated [5] thus making cfDNA quantification a feasible option to assess the extent of genotoxic damage caused by the organic solvents found in these paints. In recent years, many biological biomarkers have been used to evaluate and/or quantify the different types of oxidative stress, including DNA/RNA damage, i.e., lipid peroxidation, ROS, antioxidants and protein oxidation/nitration (Table 1) [6]. To date, cfDNA has been only applied for diagnosis and prognosis of various types of pathologies or conditions (cancer, autoimmune diseases, tuberculosis, myocardial infarction, sepsis, trauma, pregnancy, among others) [7–11], considering that its usually low concentration in blood (0–100 ng/mL) [12] and other body fluids increases significantly as a result of cellular death associated [13]. However, cfDNA may also increase in healthy individuals [14] as a result of apoptosis or necrosis of cells of the blood or other tissues [15, 16] or as a consequence of intense exercise such as the half- or ultra-marathon running [17].

**Table 1:** Biological markers of oxidative stress

Type of damage	Marker of damage
*DNA/RNA Damage*	8-hydroxyguanosine (8-OHG)
	Abasic (AP) sites
	BPDE DNA Adduct
	Double-strand DNA breaks
	Comet Assay (general DNA damage)
	UV DNA Damage (CPD, 6-4PP)
*Lipid Peroxidation*	4-Hydroxynonenal (4-HNE)
	8-iso-Prostaglandin F2alpha (8-isoprostane)
	Malondialdehyde (MDA)
	Thiobarbituric acid reacting substances (TBARS)
*Reactive Oxygen Species*	Universal ROS/RNS
	Hydrogen Peroxide
	Nitric Oxide
*Antioxidants*	Catalase
	Glutathione
	Superoxide Dismutase
	Oxygen Radical Antioxidant Capacity (ORAC)
	Hydroxyl Radical Antioxidant Capacity (HORAC)
	Total Antioxidant Capacity (TAC)
*Protein Oxidation/Nitration*	3-Nitrotyrosine
	Advanced Glycation End Products (AGE)
	Advanced Oxidation Protein Products (AOPP)
	BPDE Protein Adduct

The comet assay, a biomarker of effect, has been widely used to quantify the genotoxic damage from occupational exposure, based on the appearance of nuclear fragments -product of single- and double-strand DNA breaks- and alkali- labile sites that have migrated from the nucleus and having the appearance of comet tail whose length and DNA contents may be measured and correlated with the extent of DNA damage [5, 18, 19]. Considering that occupational exposure to organic solvents, as those contained in car paints, can lead to disease and also that there is a large population of car paint workers in Colombia who, in their majority, do not observe the rules of industrial biosecurity and therefore are exposed to them, it is necessary to implement methodologies that not only allow an early identification of adverse effects caused by these genotoxic agents, but also to monitor them after the compliance with biosafety standards by owners and workers of car paint shops.

According to the above, our efforts were aimed to evaluate cfDNA concentrations in the serum of individuals occupationally exposed to the organic solvents contained in car paints and to analyze them in the light of the results of the comet assay and the levels of indoor air organic solvents, as well as of parameters such as age, time of exposure, smoking habits and alcohol intake.

## Methods

### Study population

This was a single blind retrospective research, which involved two cohorts. One cohort was composed by 33 male gender individuals 18–73 years old, routinely exposed to car paints, who were recruited among ten handicraft car paint shops at the “7 de agosto” neighborhood in Bogota DC, Colombia. On the other hand, 33 workers employed in a hoses factory and not occupationally exposed to organic solvents, were selected to constitute the non-exposed cohort, who were recruited in another area within the same neighborhood and with similar characteristics except for the proximity to car paint shops. This research was approved by the Ethical Committee of the Universidad del Rosario.

### Selection criteria

The exposed cohort consisted of adult men being exposed to car paints for periods of at least three months. The members of the non-exposed cohort were selected using the same criteria applied for the exposed cohort except for the exposure to organic solvents and also considering that their ages were similar to the exposed group, with a maximum difference of ± 2 years.

Furthermore, we made sure that, in the case of a worker having labored in more than one shop, the biosafety conditions were similar in all the places he worked at before.

### Exclusion criteria

Individuals who had suffered from hepatitis or cancer or another severe disease, or had been under chemotherapy or radiotherapy or any other recent prolonged medical treatment were excluded as well as those who provided inconsistent personal information.

### Blood sampling

Two samples from peripheral blood were taken: one intended for lymphocytes isolation and further comet assay and the other for cfDNA assay. Sampling was carried out immediately after exposure at the end of the workweek. Samples to determine the cfDNA were collected in tubes with serum separator gel (BD 367988 Vacutainer® RST tubes for Rapid Obtaining Serum) and those for the comet assay in vacutainer tubes with heparin (Ref BD 367874 Vacutainer® Sodium Heparin). All samples were immediately transported to the laboratory within 10 min. The tubes for serum collection were centrifuged at 3000 RPM for 10 min and then transferred to eppendorf tubes, in order to ultra-centrifuge for 10 min at 14000 RPM twice. The supernatant was immediately frozen at −20 °C for up to three weeks. This procedure excluded the possibility that the supernatant had DNA content from blood cells. The blood samples for the comet assay were immediately processed for the isolation of lymphocytes. Samples of all individuals, exposed and non-exposed, were processed simultaneously.

### Lymphocytes isolation

Lymphocytes isolation was performed by density gradients with histopaque-1077 (Sigma Aldrich, St. Louis, MO, USA) and centrifugation at 2300 rpm during 30 min. Lymphocytes pellet was re-suspended in PBS 1X (Gibco, Life Technologies, Nebraska, USA), to perform the comet assay.

### Exclusion cytoxicity test

The trypan blue test (Life Technologies, Nebraska, USA) was carried out to determine that the damage to be evaluated in the cells were the result of genotoxicity and not cytotoxicity [20, 21]. The relationship between the number of live and dead lymphocytes was between 85–95 % and the volume of cell suspension used in the test was 4×10^3^ lymphocytes.

### Comet assay

The alkaline comet assay was performed according to the proposed Collins et al. protocol [22], using the Trevigen Comet Assay Kit (Trevigen, Gaithersburg, USA). A minimum of 100 cells per individual were analyzed by using fluorescence microscope (Nikon Instruments Inc, USA), with a magnification of 100×.

Three trials were performed. The first one was aimed to get positive controls using hydrogen peroxide (H_2_O_2_) as a genotoxic agent in lymphocytes; the second was conducted to evaluate cell damage due to occupational exposure to organic solvents at the car paint shops in exposed individuals and, third, to evaluate cell damage in non-exposed individuals. All assays were performed in duplicate.

The comets were classified through the Comet Score publisher program, in five categories or levels of damage according to the percentage of DNA in the tail, as follows: 0: no damage (<5 %), 1: low damage (6–25 %), 2: moderate damage (26–50 %), 3: high damage (51–75 %) and 4: severe damage (>76 %) [19, 22, 23].

In each of the sampled individuals, all types of comet were considered. For this analysis, the type of comet more often observed (mode) in each sample was used, as indicated by Moro et al. [5] and Rombaldi et al. [24].

### Cell-free DNA

cfDNA was determined in the serum collected from each blood sample, following the proposed Goldshtein et al. [25] protocol. SYBR® Gold Nucleic Acid Gel Stain 10000× (Invitrogen GmbH, Karlsruhe, Germany) was used and two dilutions were made for this purpose: first, 1:1000 in pure dimethyl sulfoxide (DMS) (Sigma-Aldrich) and second, 1:8 in PBS (1×).

As concentration control, a calibration curve was constructed with known concentrations of salmon sperm DNA. Fluorescence was measured in the fluorometer (Tecan, Männedorf, Switzerland). The wavelength excitation was recorded at 485 nm and the emission wavelength at 535 nm by using data analysis software, Magellan v7.1 (Tecan Genius). These results were then confirmed by spectrophotometry (Thermo Scientific NanoDrop 2000 Series 3248, MA, USA).

cfDNA concentrations in the serum samples were calculated from the interpolation of the data obtained from the calibration curve of DNA standards. To confirm the specificity of the assay and to eliminate the possible influence of serum in the results, 10 samples of the extracted serum were randomly selected and incubated with DNAse I (500 U/mL) (Thermo Scientific, USA) at 37 °C for 5 h and used as negative control. Values of fluorescence thus obtained were then subtracted from those corresponding to the serum samples which in turn had previously been incubated with SYBR® Gold Nucleic Acid Gel Stain 10000× (Invitrogen GmbH, Karlsruhe, Germany).

The cfDNA concentrations obtained were classified into three categories or levels, according to the following reference values: low: 0–580 ng/mL; medium: 581–2500 ng/mL; high: >2500 ng/mL [25].

### Determination of benzene, toluene and xylene (BTX) in air samples

Prior establishment of the risks map within the workshops, stationary air sampling was carried out through active sampling tubes, placed at 1,5 meter height and in the middle of the hall, with the aspiration flow fixed at 0,2 liters/minute, according to the “National Institute for Occupational Safety and Health” (NIOSH) analytical method 1500 for aromatic hydrocarbons [26] and then quantitative determination of BTX was performed. The reference Threshold Limit Values were those indicated by the American Industrial Hygienists Conference (ACGIH) [27].

### Statistical analysis

The results were analyzed using the SPSS v22.0 (Statistical Package to Social Scientific) program. Homogeneity of variances was evaluated by the Levene’s test and normality by the Shapiro-Wilk and the Lilliefors corrected Kolmogorov-Smirnov tests.

With the purpose to reduce the effect of age as a possible confounding factor, we paired 1:1 by age the exposed and the non-exposed individuals. The asymptotic or exact McNemar’s test (binomial, expected values < 5) for paired samples was used to evaluate significant differences in tabaquism and alcohol intake.

The nonparametric Wilcoxon exact test for two related samples was performed to search possible significant differences in the frequencies of the type of comets as well as in cfDNA concentrations between the two cohorts. To compare the frequencies of the higher categories of comets between the two groups, an exact McNemar’s test was applied. In addition, we used Spearman rank correlation to test the correlation between cfDNA concentrations and type of comets, and between exposure time and extent of DNA damage (assessed by comet assay and cfDNA) as well. To compare the extent of genotoxic damage among the visited workshops, the exact Kruskal-Wallis test was applied, excluding from analysis those having one worker exposed. Furthermore, we evaluated possible significant differences in the extent of DNA damage related to exposure time, and also BTX concentrations among car paint shops, applying the exact Kruskal-Wallis test. *P*-value less than 5 % (*p* < 0,05*) and 1 % (*p* < 0,01**) was considered as statistically significant.

## Results

The exposed and non-exposed cohorts were comparable from a statistical point of view, which was the result of an experimental design consisting in a pairing 1:1 by age and also whereas no significant differences were found in smoking habits or alcohol intake (*p* = 0,687 and *p* = 0,219 respectively, exact McNemar’s test for paired samples) (Table 2).

**Table 2:** Epidemiologic characterization of the cohorts

Characteristics	Exposed (*N* = 33)	Non-exposed (*N* = 33)	Significance
Ages (mean ± SD)	46,18 ± 14.59	46,18 ± 14.68	1,000
Time of exposure in months (mean ± SD)	234,33 ± 141,38 (median = 212,00)		
Smokers	5 (15,2 %)	7 (21,2 %)	0,687 (e)
Alcohol intake	32 (97,0 %)	28 (84,8 %)	0,219 (e)

*SD* standard deviation

### Comet assay and cfDNA

**Fig. 1:**
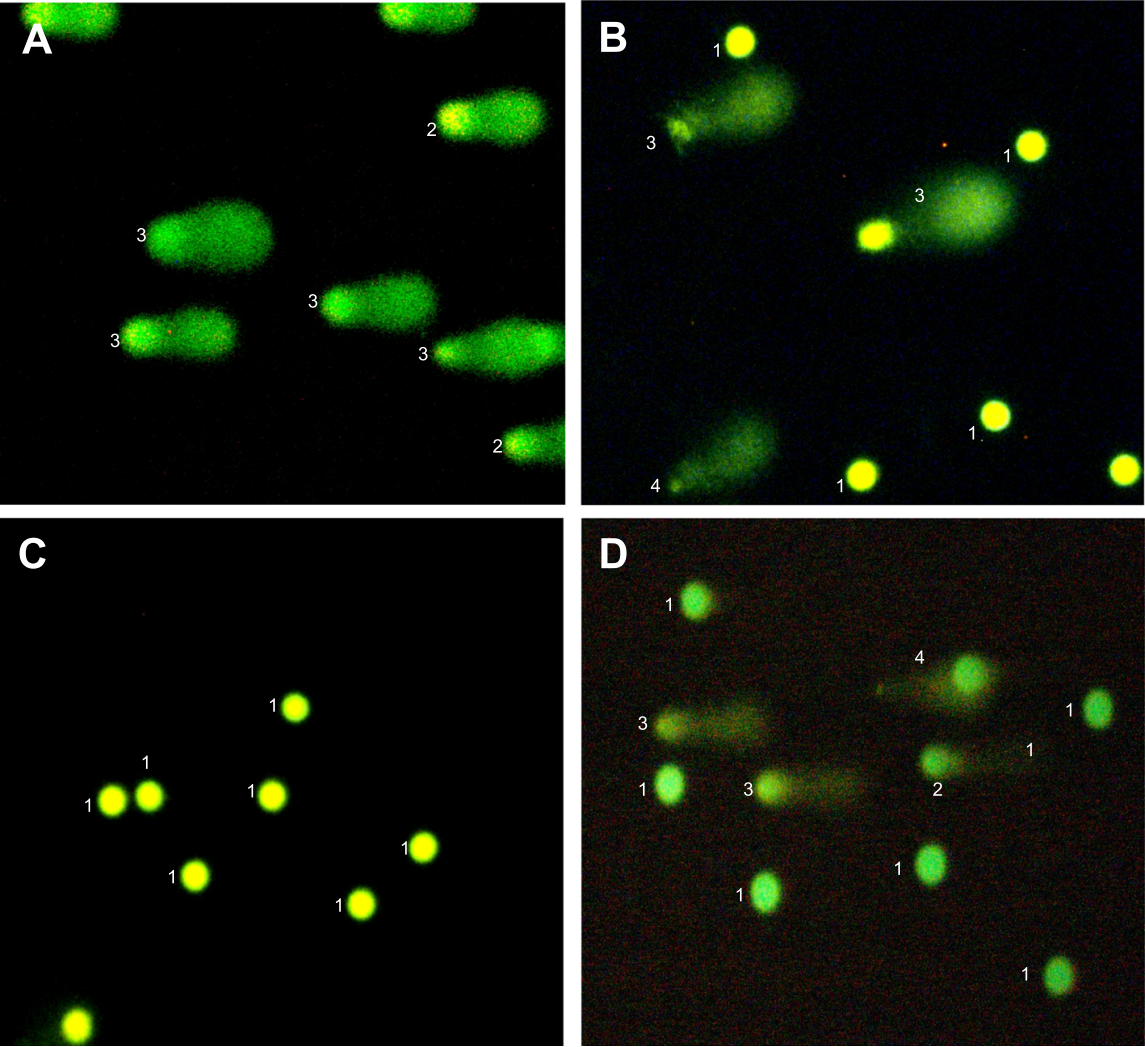
Types of comet obtained from the alkaline comet assay. **a** Positive control. Predominance of type 3 is observed, which means that over 50 % of the DNA has migrated from the core. Magnification: 10×. **b** Exposed individual who had comets type 1, 3, and 4, although type 3 was the predominant after making the total count. Magnification: 10×. **c** Non-exposed individual. The observable comets correspond entirely to type 1, where less than 25 % of the DNA has migrated. Magnification: 10×. **d** In this exposed individual, comets type 1, 2, 3 and 4 are observable, being type 4 the predominant after making the total count. Magnification: 10×

Similarly to that happened with the positive controls (Fig. 1a), 66,7 % of the group exposed to solvents showed type 3 and 4 comets (Table 3), in which over 50 % of total DNA was fragmented and located outside the nucleus (Fig. 1b), versus the non-exposed group, in which 82 % had type 1 comets with less than 15 % of the DNA outside the nucleus (Fig. 1c), which means that exposure to solvents has a statistically significant genotoxic effect over the exposed individuals (*p* < 0,001, Wilcoxon exact one-sided test). Frequencies of comets 3 and 4 in the exposed (66,7 %) were significantly higher than in the non-exposed ones (9,1 %) (*p* < 0,001, Exact McNemar’s test).

**Table 3:** Distribution of comets in the exposed and the non-exposed cohorts

Type of comets	Exposed % (N)	Non-exposed % (N)
0	0	0
1	21,2 (7)*	81,8 (27)*
2	12,1 (4)	9,1 (3)
3	45,5 (15)**	9,1 (3) **
4	21,2 (7)	0

**p < 0,01, *p < 0,05

In addition, workers employed in the #4 and #9 car paint shops had significantly higher genotoxic damage, evaluated by comet assay, compared to the other workshops (*p* = 0,025, Exact Kruskal-Wallis Test) (Additional file 1: Table S1). Having compared BTX concentrations between workshops and taking into account that It was not possible to measure them in the workshop #10, we found significant differences (*p* < 0,001, Exact Kruskal-Wallis Test). Thus, while benzene levels in workshops 1, 2, 4 and 7, and toluene levels in workshops 2, 4 and 9 were significantly higher, in turn, the distribution of the toluene levels was higher in workshops 2 and 4, followed by 7 and 9. Once compared the extent of genotoxic damage, assessed by comet assay, and the exposure time between workshops, we did not find significant differences (*p* = 0,456, Exact Kruskal-Wallis Test) (Additional file 1: Table S1).

With respect to the cfDNA quantification, its concentrations in the exposed were significantly higher than in the non-exposed individuals (*p* < 0,001, Wilcoxon exact one-sided test) (Table 4). After having rated each individual concentration (exposed and non-exposed) as low, medium or high, according to the reference values and the percentage of subjects in each category (Table 5), we found significantly higher cfDNA levels in the exposed cohort (*p* = 0,016, Wilcoxon exact one-sided test). Moreover, a significant positive correlation between the alcohol intake time of exposed individuals and the cfDNA concentration was established (*r* = 0,346, *p* = 0,033, Spearman Rank Correlation) and also with the extent of DNA damage evaluated by the type of comet (*r* = 0,310, *p* = 0,047, Spearman rank correlation). However, there was no correlation between cfDNA concentration and type of comet (*r* = 0,084, *p* = 0,641, Spearman rank correlation). In turn, a significant positive correlation between the alcohol intake time and the cfDNA concentration in non-exposed individuals was found, but not so with the alcohol intake time and the extent of DNA damage assessed by type of comet (*r* = -0,085, *p* = 0,687, Spearman rank correlation). Similarly to that happened in the exposed individuals, there was no correlation between cfDNA concentration and type of comet in the non-exposed (*r* = 0,081, *p* = 0,655, Spearman rank correlation). On the other hand, there were no significant differences in the extent of genotoxic damage assessed by cfDNA concentration or their corresponding ranges between the car paint shops (*p* = 0,297, Exact Kruskal-Wallis Test) (Additional file 1: Table S1).

**Table 4:** cfDNA concentrations (ng/mL) in the exposed and the non-exposed cohorts

	Exposed	Non-exposed
Mean	2398,90	1301,83
*Minimum value*	*63*	*0*
*Maximal value*	*5159*	*3957*
Standard deviation	1513,28	1276,32
Median	1991,00	994,00
N	33	33

**Table 5:** cfDNA levels in the exposed and the non-exposed cohorts

cfDNA levels (ng/mL)	Exposed % (N)	Non-exposed % (N)
Low (0–580)^a^	9,1 (3)	30,3 (10)
Medium (581–2500)^a^	57,6 (19)	51,5 (17)
High (>2500)^a^	33,3 (11)	18,2 (6)

^a^Reference values

### Association between DNA damage and exposure time

**Fig. 2:**
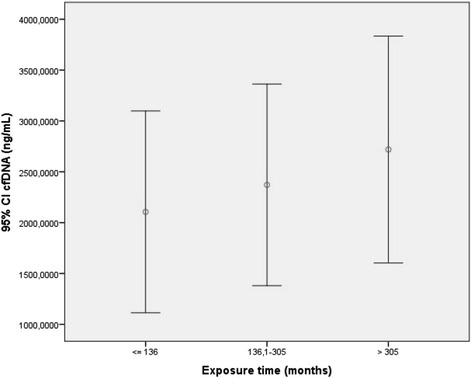
Mean and 95 % confidence interval of cfDNA concentrations by range of exposure time in the exposed cohort. Exposure time to airborne solvent vapors significantly increased the extent of DNA damage assessed by cfDNA concentration (*r* = 0,28, *p* = 0,053, Spearman rank correlation)

There was a moderate significant positive relationship between the extent of DNA damage represented by comets 3 and 4 and the exposure time to indoor airborne solvent vapors (*r* = 0,317, *p* = 0,047). Similar results were found between cfDNA concentrations and the exposure time (*r* = 0,28, *p* = 0,053, Spearman rank correlation) (Fig. 2).

It is important to mention that, in the car paint shops, the procedures used in the task of preparing mixtures for paints and applying to vehicles tend to be traditional and therefore have not changed significantly in the last 20 years. Consequently, no one had made any effort to applying any biosafety protocol in these places, so that compliance with personal protective devices was very poor. These are noteworthy facts, since the painters had been successively employed in various workshops in the same sector, which had similar features to those of their current workplace. In spite of BTX concentrations in the indoor air of the car paint shops were determined (Additional file 1: Table S1), we could not standardize the working time and daily exposure to these solvents because they varied according to workload, thus making impossible to assess the exposure of a typical handicraft car painter based on existing data, as those reported from car painting workshops belonging to the formal industrial sector, in which rights, schedules and biosafety regulations are abided and respected.

## Discussion

The vast majority of paint shops of motor vehicles in Bogotá and other cities in Colombia lack adequate ventilation and personal protective equipment. For this reason painters are permanently exposed to organic solvents during all the operations involved, such as sanding of the surface, cleansing, masking, varnish preparation and spraying, activities that entail serious risks, because of their mutagenic and carcinogenic properties. Various methods are used to date to evaluate cell damage caused by exposure to these xenobiotic agents, such as the cytogenetic and the micronucleus assays. In view of that these techniques are wasteful because of its costs and long-term analysis, in this research we conducted a new field trial evaluation of genotoxicity, based on the quantification of cfDNA in serum. This method is advantageous, not only because of its minimally invasive characteristics but also due to cfDNA stability in serum and to its easy accessibility. In addition, we used the comet assay as an alternative method for evaluating the extent of DNA damage.

Our results showed significant differences in the frequency of higher categories of comets (*p* < 0,001) and in cfDNA concentrations (*p* < 0,001) in the exposed cohort, in comparison with those observed in non-exposed (control group) (Tables 3 and 4). However, although there was a significant statistical difference in the mean of cfDNA between individuals exposed and non-exposed, these concentrations grouped into ranges or levels did not appear to be as discriminatory, it is also true the fact that in our research we tried where possible to minimize the influence of confounding factors between the two groups, such as age, degree of physical activity associated with the occupation as well as exercise habits, alcohol intake and smoking.

Although the exposed and non-exposed distribution of cfDNA concentrations in the medium level (581–2500 ng/mL) was very similar between, significant differences were observed in the distribution of the high (>2500 ng/mL) cfDNA level (Table 4), where the number of exposed was greater than the non-exposed. In the low (0–580 ng/ml) cfDNA level, there was a significant statistical difference in favor of the non-exposed individuals, which were in a greater number than the exposed, fact that can be explained when considering that individuals belonging to the control group were not exposed to organic solvents and in consequence a less extent of DNA damage could be reasonable to wait.

The wide interindividual variability in cfDNA values observed in both groups (exposed and non-exposed) (Table 4), might be explained by differences in the extent of tissue damage - which may vary according to age, exposure time, workload or BTX levels at workshops – but also by differences in the oxidative metabolism of genotoxic agents, which could affect cfDNA concentrations. In the case of car painters, the exposure to those agents was obvious while in the control group, one could think in other environmental genotoxic agents e.g. carbon dioxide, alcohol, or those contained in certain products used for home cleansing. These results are in agreement with previous studies where wide variability in the mean of cfDNA concentrations was also observed [7, 28, 29]. It should be noted also that the kinetics of genotoxic damage-related cfDNA release into the bloodstream and its subsequent clearance have not yet elucidated [30] thus making the cfDNA, as rightly Danese et al. said, a “hard to read” analyte [31].

In addition, it is noteworthy that though a significant Spearman coefficient of correlation between the cfDNA content and the DNA damage (assessed by comet assay) was not observed, both parameters reflect the extent of the genotoxic damage from exposure to solvents. This finding could be explained by an adaptive response of the organism addressed to the effective cfDNA elimination from blood but also to a survival mechanism of cells with damaged DNA [32]. In fact, it has been recently reported an enhancement of DNA damage in lymphocytes along with a cfDNA content reduction, in human occupational exposure to low-dose gamma-neutron and tritium β-radiation [32].

On the other hand, occupational exposure time to organic solvents in car paint shops significantly increased the risk of genetic damage (Fig. 2), thus accomplishing the Bradford-Hill dose-response relationship criteria for causality, which means there is a valid causal connection between exposure time to airborne solvent vapors and extent of DNA damage [33].

Although cfDNA concentration can vary depending on multiple factors, including degree of exercise, the increase that has been observed after exhaustive exercise such as marathon disappears within two hours after the race [17, 34]. Consequently, activities conducted during the day could not be associated with increased concentrations of cfDNA, taking into account that the occupational-associated degree of physical activity in the two groups, exposed and non-exposed, was similar. Thus, the control group was composed of workers of a hoses factory, occupation that demands similar physical effort to that of workers in the car paint shops. In addition, none of them, exposed or non-exposed, reported exercising regularly or other possible sources of cfDNA (e.g. infection, trauma, inflammatory disease or cancer).

Considering our results together with the understanding that apoptotic and necrotic cells are the main source of cfDNA and also that its concentration increases due to an augmented vulnerability of damaged cells to cell death [35], it is possible to assume that the cfDNA is a product of cellular DNA damage. Consequently, determination of cfDNA could be taken into account along with other biomarkers, in order to support the diagnostic of genotoxicity in individuals occupationally exposed to organic solvents in car paint shops, for which this could be a useful tool, just as it has been for patients suffering from inflammatory or infectious processes, autoimmune diseases and cancer, among others [28].

The increase in DNA damage in the exposed cohort can be explained based on the oxidative metabolism of BTX, wherein the intermediate metabolites may give rise to reactive oxygen species (ROS) which, in turn, oxidize the DNA. Furthermore, these metabolites can produce DNA adducts, generating DNA modifications, such as alkali-labile sites, single-stranded breaks (SSB) or double strand breaks (DSB) [19].

Despite of the cfDNA test has been commonly used to evaluate the progression of neoplastic disease [10, 36] and also to study its relationship with apoptosis [37], using techniques such as PCR, UV-visible spectrophotometry and the newly discovered fluorometric, so far there are no reports of its application in assessing the damage generated by the organic solvents that are used to manufacture car paintings. While clinical significance of cfDNA has not been fully elucidated, the results of our research are of interest, because they allowed establishing significant differences between the cfDNA levels in the serum of individuals occupationally exposed to solvents compared to the non-exposed ones.

The high frequency of DNA damage observed in this research indicates an urgent need to implement methodologies that not only allow an early identification of adverse effects caused by these genotoxic agents, but also to monitor them after the compliance with biosafety standards by owners and workers of car paint shops.

## Conclusions

Our results showed that car painters had a significant increase in the cfDNA circulating in the serum, which is an evidence of genetic damage caused by occupational exposure to organic solvents, regardless that the corresponding levels in air were or not within the allowable limits, results that should be analyzed by the appropriate control agencies in order to redefining the permissible concentrations of solvents in air. In the light of the IARC reports classifying work vehicle painting as a carcinogenic industrial process, it is clear that occupational exposure to organic solvents contained therein constitutes a public health problem in Colombia, which reiterates the need to continuously monitor them and monitor adherence to relevant biosecurity rules, as well. The feasibility of making the cfDNA test in serum as a part of the job entrance examinations and monitoring of workers in the researched labor sector could be one of the most important potential applications of the findings of our research in the field of occupational health.

## Abbreviations

BTX, benzene, toluene, xylene, trimethylbenzene; cfDNA, cell-free DNA; DSB, double strand breaks; ROS, reactive oxygen species; SSB, single-stranded breaks

## Additional file

Additional file 1: Table S1. cfDNA concentrations and type of comet in the exposed individuals - grouped by car paint shops - and BTX concentrations in the indoor air. **Table S2.** Socio-demographic data of the exposed cohort. **Table S3.** Socio-demographic data of the non-exposed cohort. **Table S4.** Exposed cohort. cfDNA and total count and types of comet. **Table S5.** Non-exposed cohort. cfDNA and total count and types of comet. **Table S6.** Air borne solvents concentrations in workshops. **Table S7.** Exposed cohort. Comet score data. **Table S8.** Non-exposed cohort. Comet score data. (DOCX 73 kb)

## References

[1] WHO-IARC. Painting, Firefighting, and Shifwork/IARC Monographs on the Evaluation of the Carcinogenic Risks of Chemicals to Humans. Occupational exposure as a painter. 2010. https://monographs.iarc.fr/ENG/Monographs/vol98/mono98.pdf. Accessed 01 Sept 2015.

[2] Lan Q, Zhang L, Li G, Vermeulen R, Weinberg RS, Dosemeci M (2004). Hematotoxicity in workers exposed to low levels of benzene. Science.

[3] WHO-IARC. A review of human carcinogens. Part F: Chemical agents and related occupations/IARC Working group on the evaluation of carcinogenic risks to humans. 2012. http://monographs.iarc.fr/ENG/Monographs/vol100F/mono100F.pdf. Accessed 01 Sept 2015. PMC4781612 23189753

[4] Velandia-Neira E (2004). Velocidad de conducción nerviosa en trabajadores que manejan solventes orgánicos. Salud Trabajo y Ambiente.

[5] Moro AM, Brucker N, Charao M, Bulcao R, Freitas F, Baierle M (2012). Evaluation of genotoxicity and oxidative damage in painters exposed to low levels of toluene. Mutat Res.

[6] Ho E, Karimi Galougahi K, Liu CC, Bhindi R, Figtree GA (2013). Biological markers of oxidative stress: Applications to cardiovascular research and practice. Redox Biol.

[7] Czeiger D, Shaked G, Eini H, Vered I, Belochitski O, Avriel A (2011). Measurement of circulating cell-free DNA levels by a new simple fluorescent test in patients with primary colorectal cancer. Am J Clin Pathol.

[8] Rodriguez-Arnaiz R. Las toxinas ambientales y la genetica. In: Las toxinas ambientales y sus efectos genéticos. Fondo de Cultura Económica, México DF, México. 1995. http://www.biblioises.com.ar/. Accessed 19 Feb 2015.

[9] Roth C, Pantel K, Muller V, Rack B, Kasimir-Bauer S, Janni W (2011). Apoptosis-related deregulation of proteolytic activities and high serum levels of circulating nucleosomes and DNA in blood correlate with breast cancer progression. BMC Cancer.

[10] Schwarzenbach H, Hoon DS, Pantel K (2011). Cell-free nucleic acids as biomarkers in cancer patients. Nat Rev Cancer.

[11] Stroun M, Maurice P, Vasioukhin V, Lyautey J, Lederrey C, Lefort F (2000). The origin and mechanism of circulating DNA. Ann N Y Acad Sci.

[12] Parekh H, Dashora P, Acharya A, Vaniawala S, Bapat A, Mukhopadhyaya P (2015). Suspended in blood and circulating within, cell free (cf) DNA connects with a vast range of adverse human health conditions including cancer: A review. Res J Pharm, Biol Chem Sci.

[13] Anker P, Stroun M (2000). Circulating DNA in plasma or serum. Medicina.

[14] Breitbach S, Tug S, Simon P (2012). Circulating cell-free DNA: an up-coming molecular marker in exercise physiology. Sports Med.

[15] van der Vaart M, Pretorius PJ (2008). Circulating DNA. Its origin and fluctuation. Ann N Y Acad Sci.

[16] Jahr S, Hentze H, Englisch S, Hardt D, Fackelmayer FO, Hesch RD (2001). DNA fragments in the blood plasma of cancer patients: quantitations and evidence for their origin from apoptotic and necrotic cells. Cancer Res.

[17] Atamaniuk J, Stuhlmeier KM, Vidotto C, Tschan H, Dossenbach-Glaninger A, Mueller MM (2008). Effects of ultra-marathon on circulating DNA and mRNA expression of pro- and anti-apoptotic genes in mononuclear cells. Eur J Appl Physiol.

[18] de Oliveira HM, Dagostim GP, da Silva AM, Tavares P, da Rosa LA, de Andrade VM (2011). Occupational risk assessment of paint industry workers. Indian J Occup Environ Med.

[19] Swanepoel A. Evaluation of DNA damage and DNA repair by the comet assay in workers exposed to organic solvents: North-West University; 2004. http://dspace.nwu.ac.za/handle/10394/1482. Accessed 01 Sept 2015.

[20] Fenech M, Holland N, Chang WP, Zeiger E, Bonassi S (1999). The HUman MicroNucleus Project--An international collaborative study on the use of the micronucleus technique for measuring DNA damage in humans. Mutat Res.

[21] Pitarque M, Vaglenov A, Nosko M, Hirvonen A, Norppa H, Creus A (1999). Evaluation of DNA damage by the Comet assay in shoe workers exposed to toluene and other organic solvents. Mutat Res.

[22] Collins AR, Dusinska M, Horska A (2001). Detection of alkylation damage in human lymphocyte DNA with the comet assay. Acta Biochim Pol.

[23] Moller P, Knudsen LE, Loft S, Wallin H (2000). The comet assay as a rapid test in biomonitoring occupational exposure to DNA-damaging agents and effect of confounding factors. Cancer Epidemiol Biomarkers Prev.

[24] Rombaldi F, Cassini C, Salvador M, Saffi J, Erdtmann B (2009). Occupational risk assessment of genotoxicity and oxidative stress in workers handling anti-neoplastic drugs during a working week. Mutagenesis.

[25] Goldshtein H, Hausmann MJ, Douvdevani A (2009). A rapid direct fluorescent assay for cell-free DNA quantification in biological fluids. Ann Clin Biochem.

[26] (NIOSH) TNIfOSaH. CDC Centers for Disease Control and Prevention. CDC. 2015. http://www.cdc.gov/niosh/docs/2003-154/pdfs/1500.pdf. Accessed 07 April 2015.

[27] ACGIH. Industrial Hygiene, Environmental, Occupational Health & Safety Resource. ACGIH. 2015. http://www.acgih.org/. Accessed 01 Feb 2016.

[28] Gormally E, Caboux E, Vineis P, Hainaut P (2007). Circulating free DNA in plasma or serum as biomarker of carcinogenesis: practical aspects and biological significance. Mutat Res.

[29] Salazar-Jordán H, García-Robayo DA, Amaya J, Castillo M, Briceño I, Aristizábal F (2009). Cuantificación de ADN libre en plasma sanguíneo de voluntarios sanos en una población bogotana. NOVA.

[30] Al-Humood S, Zueriq R, Al-Faris L, Marouf R, Al-Mulla F (2014). Circulating cell-free DNA in sickle cell disease: is it a potentially useful biomarker?. Arch Pathol Lab Med.

[31] Danese E, Minicozzi AM, Benati M, Montagnana M, Paviati E, Salvagno GL (2015). Comparison of genetic and epigenetic alterations of primary tumors and matched plasma samples in patients with colorectal cancer. PLoS One.

[32] Korzeneva IB, Kostuyk SV, Ershova LS, Osipov AN, Zhuravleva VF, Pankratova GV (2015). Human circulating plasma DNA significantly decreases while lymphocyte DNA damage increases under chronic occupational exposure to low-dose gamma-neutron and tritium beta-radiation. Mutat Res.

[33] Hill AB (1965). The environment and disease: association or causation?. Proc R Soc Med.

[34] Atamaniuk J, Vidotto C, Tschan H, Bachl N, Stuhlmeier KM, Muller MM (2004). Increased concentrations of cell-free plasma DNA after exhaustive exercise. Clin Chem.

[35] Pollack M, Leeuwenburgh C (2001). Apoptosis and aging: role of the mitochondria. J Gerontol A Biol Sci Med Sci.

[36] Garcia-Olmo DC, Picazo MG, Toboso I, Asensio AI, Garcia-Olmo D (2013). Quantitation of cell-free DNA and RNA in plasma during tumor progression in rats. Mol Cancer.

[37] Kaminski BC, Grabenbauer GG, Sprung CN, Sauer R, Distel LV (2006). Inter-relation of apoptosis and DNA double-strand breaks in patients with multiple primary cancers. Eur J Cancer Prev.

