# Supplementary material for: Quantification of cell-free DNA for evaluating genotoxic damage from occupational exposure to car paints
Source: J Occup Med Toxicol. 2016 Jul 15;11:33. doi: 10.1186/s12995-016-0123-8 (PMC4946235; doi:10.1186/s12995-016-0123-8)
Supplement: Additional file 1: Table S1. — cfDNA concentrations and type of comet in the exposed individuals - grouped by car paint shops - and BTX concentrations in the indoor air. Table S2. Socio-demographic data of the exposed cohort. Table S3. Socio-demographic data of the non-exposed cohort. Table S4. Exposed cohort. cfDNA and total count and types of comet. Table S5. Non-exposed cohort. cfDNA and total count and types of comet. Table S6. Air borne solvents concentrations in workshops. Table S7. Exposed cohort. Comet score data. Table S8. Non-exposed cohort. Comet score data. (DOCX 73 kb) [file 12995_2016_123_MOESM1_ESM.docx]

**Supplementary Material**

**Supplementary Table 1. cfDNA concentrations and type of comet in the exposed individuals - grouped by car paint shops - and BTX concentrations in the indoor air**

| **Car paint shops** | **N** | **Exposure time** (months) | **Airborne BTX concentration** (mg/L) | | | **Type of comet** | **[cfDNA]** (ng/mL) | |
| --- | --- | --- | --- | --- | --- | --- | --- | --- |
|  |  |  | **Benzene** | **Toluene** | **Xylene** |  |  |  |
| 1 | 7 | 264 | 0,330 | 10,74 | 28,92 | 3 | 4414 | Mean:3213,20  DS:1281,50  Median:2675,00 |
|  | 8 | 168 |  |  |  | 3 | 2376 |  |
|  | 9 | 424 |  |  |  | 2 | 4736 |  |
|  | 13 | 228 |  |  |  | 2 | 1865 |  |
|  | 14 | 190 |  |  |  | 1 | 2675 |  |
| 2 | 10 | 324 | 0,310 | 27,22 * | 115,20 * | 3 | 4700 | Mean: 2329,33  DS: 2320,25  Median: 2225,00 |
|  | 11 | 74 |  |  |  | 2 | 2225 |  |
|  | 12 | 86 |  |  |  | 2 | 63 |  |
| 4 | 16 | 240 | 0,300 | 25,27* | 101,54* | 3 | 5195 | Mean: 3287,00  DS: 2028,54  Median:3410,50 |
|  | 17 | 204 |  |  |  | 3 | 1132 |  |
|  | 18 | 420 |  |  |  | 4 | 4830 |  |
|  | 19 | 540 |  |  |  | 3 | 1991 |  |
| 7 | 22 | 132 | 0,340 | 8,25 | 70,12 | 1 | 2709 | Mean: 1955,66  DS: 843,08  Median: 2113,00 |
|  | 23 | 144 |  |  |  | 1 | 1045 |  |
|  | 24 | 384 |  |  |  | 1 | 2113 |  |
| 8 | 25 | 384 | 0,200 | 12,80 | 35,70 | 1 | 1567 | Mean: 1547,40  DS: 472,61  Median: 1567,00 |
|  | 26 | 360 |  |  |  | 3 | 2220 |  |
| 9 | 27 | 384 | 0,100 | 14,50 | 67,30 | 3 | 323 | Mean: 1974,00  DS: 1854,29  Median: 1504,00 |
|  | 28 | 504 |  |  |  | 4 | 4565 |  |
|  | 29 | 120 |  |  |  | 1 | 1970 |  |
|  | 30 | 72 |  |  |  | 4 | 1038 |  |
|  | 31 | 348 |  |  |  | 3 | 1118 |  |
|  | 32 | 360 |  |  |  | 3 | 1749 |  |
|  | 33 | 48 |  |  |  | 3 | 1083 |  |

* Permissible limits of BTX concentrations in air (Threshold Limit Values TLV) [20]

** Car paint shops 3, 5 and 6 were not taken into account because of their number of workers, which was equal to 1

**Supplementary Table 2. Socio-demographic data of the exposed cohort**

| **Car paint shop** | **Exposed** | | | | |
| --- | --- | --- | --- | --- | --- |
|  | **N°** | **Socio-demographic data** | | | |
|  |  | **Age** | **Exposure time** (months) | **Smoking 1:Yes 2:No** | **Alcohol intake 1:Yes 2:No** |
| 10 | **1** | 43 | 132 | 2 | 2 |
|  | **2** | 34 | 96 | 2 | 1 |
|  | **3** | 21 | 13 | 2 | 1 |
|  | **4** | 31 | 37 | 1 | 1 |
|  | **5** | 39 | 120 | 2 | 1 |
|  | **6** | 50 | 193 | 2 | 1 |
| 1 | **7** | 53 | 264 | 2 | 1 |
|  | **8** | 33 | 168 | 2 | 1 |
|  | **9** | 58 | 424 | 2 | 1 |
| 2 | **10** | 57 | 228 | 2 | 1 |
|  | **11** | 26 | 190 | 1 | 1 |
|  | **12** | 24 | 324 | 1 | 1 |
| 1 | **13** | 51 | 74 | 2 | 1 |
|  | **14** | 44 | 86 | 2 | 1 |
| 3 | **15** | 47 | 264 | 2 | 1 |
| 4 | **16** | 34 | 240 | 2 | 1 |
|  | **17** | 60 | 204 | 2 | 1 |
|  | **18** | 61 | 420 | 2 | 1 |
|  | **19** | 73 | 540 | 2 | 1 |
| 5 | **20** | 45 | 264 | 2 | 1 |
| 6 | **21** | 50 | 212 | 2 | 1 |
| 7 | **22** | 35 | 132 | 2 | 1 |
|  | **23** | 31 | 144 | 1 | 1 |
|  | **24** | 55 | 384 | 2 | 1 |
| 8 | **25** | 53 | 384 | 2 | 1 |
|  | **26** | 53 | 360 | 2 | 1 |
| 9 | **27** | 57 | 384 | 2 | 1 |
|  | **28** | 67 | 504 | 2 | 1 |
|  | **29** | 28 | 120 | 1 | 1 |
|  | **30** | 62 | 72 | 2 | 1 |
|  | **31** | 54 | 348 | 2 | 1 |
|  | **32** | 73 | 360 | 2 | 1 |
|  | **33** | 22 | 48 | 2 | 1 |

**Supplementary Table 3. Socio-demographic data of the non-exposed cohort**

| **Non-exposed** | | | |
| --- | --- | --- | --- |
| **N°** | **Socio-demographic data** | | |
|  | **Age** | **Smoking 1:Yes 2:No** | **Alcohol intake 1:Yes 2:No** |
| **1** | 42 | 2 | 1 |
| **2** | 36 | 2 | 1 |
| **3** | 20 | 2 | 1 |
| **4** | 31 | 2 | 1 |
| **5** | 37 | 2 | 1 |
| **6** | 49 | 1 | 1 |
| **7** | 51 | 2 | 2 |
| **8** | 35 | 2 | 1 |
| **9** | 59 | 2 | 1 |
| **10** | 55 | 2 | 1 |
| **11** | 25 | 2 | 1 |
| **12** | 24 | 1 | 1 |
| **13** | 52 | 1 | 1 |
| **14** | 44 | 2 | 1 |
| **15** | 47 | 2 | 1 |
| **16** | 35 | 2 | 1 |
| **17** | 58 | 2 | 2 |
| **18** | 61 | 2 | 2 |
| **19** | 72 | 2 | 2 |
| **20** | 45 | 2 | 1 |
| **21** | 49 | 2 | 1 |
| **22** | 34 | 2 | 1 |
| **23** | 32 | 1 | 1 |
| **24** | 57 | 2 | 1 |
| **25** | 51 | 2 | 1 |
| **26** | 55 | 2 | 1 |
| **27** | 59 | 2 | 2 |
| **28** | 69 | 1 | 1 |
| **29** | 30 | 1 | 1 |
| **30** | 61 | 2 | 1 |
| **31** | 56 | 1 | 1 |
| **32** | 73 | 2 | 1 |
| **33** | 20 | 2 | 1 |

**Supplementary Table 4. Exposed cohort. cfDNA and total count and types of comet**

| **Exposed** | | | | | | |
| --- | --- | --- | --- | --- | --- | --- |
| **Car paint shop** | **N°** | **cfDNA** | **Types of comet** | | | |
|  |  |  | **Type 1** | **Type 2** | **Type 3** | **Type 4** |
| 10 | 1 | 4758 | 25 | 22 | 14 | 39 |
|  | 2 | 1936 | 30 | 10 | 10 | 50 |
|  | 3 | 1303 | 47 | 6 | 13 | 34 |
|  | 4 | 1364 | 35 | 20 | 7 | 38 |
|  | 5 | 4717 | 27 | 20 | 30 | 23 |
|  | 6 | 571 | 30 | 15 | 10 | 45 |
| 1 | 7 | 4414 | 37 | 7 | 45 | 11 |
|  | 8 | 2376 | 34 | 16 | 40 | 10 |
|  | 9 | 4736 | 36 | 40 | 15 | 9 |
| 2 | 10 | 4700 | 32 | 26 | 33 | 9 |
|  | 11 | 2225 | 36 | 56 | 2 | 6 |
|  | 12 | 63 | 36 | 47 | 7 | 10 |
| 1 | 13 | 1865 | 30 | 33 | 20 | 17 |
|  | 14 | 2675 | 39 | 24 | 17 | 20 |
| 3 | 15 | 1089 | 20 | 21 | 33 | 26 |
| 4 | 16 | 5195 | 28 | 26 | 30 | 16 |
|  | 17 | 1132 | 22 | 21 | 44 | 13 |
|  | 18 | 4830 | 27 | 19 | 14 | 40 |
|  | 19 | 1991 | 28 | 5 | 52 | 15 |
| 5 | 20 | 2298 | 18 | 29 | 31 | 22 |
| 6 | 21 | 3426 | 26 | 12 | 36 | 26 |
| 7 | 22 | 2709 | 60 | 7 | 20 | 13 |
|  | 23 | 1045 | 49 | 23 | 11 | 17 |
|  | 24 | 2113 | 53 | 26 | 6 | 15 |
| 8 | 25 | 1567 | 31 | 27 | 28 | 14 |
|  | 26 | 2220 | 18 | 30 | 36 | 16 |
| 9 | 27 | 323 | 9 | 38 | 41 | 12 |
|  | 28 | 4565 | 23 | 10 | 17 | 50 |
|  | 29 | 1970 | 59 | 11 | 16 | 14 |
|  | 30 | 1038 | 17 | 13 | 25 | 45 |
|  | 31 | 1118 | 26 | 21 | 41 | 12 |
|  | 32 | 1749 | 20 | 4 | 54 | 22 |
|  | 33 | 1083 | 19 | 23 | 38 | 20 |

**Supplementary Table 5. Non-exposed cohort. cfDNA and total count and types of comet**

| **Non-exposed** | | | | | |
| --- | --- | --- | --- | --- | --- |
| **N°** | **cfDNA** | **Types of comet** | | | |
|  |  | **Type 1** | **Type 2** | **Type 3** | **Type 4** |
| **1** | 13 | 52 | 29 | 14 | 5 |
| **2** | 1018 | 60 | 24 | 2 | 14 |
| **3** | 744 | 25 | 54 | 16 | 5 |
| **4** | 3521 | 28 | 27 | 39 | 6 |
| **5** | 3611 | 50 | 34 | 5 | 11 |
| **6** | 1213 | 41 | 38 | 13 | 8 |
| **7** | 1306 | 47 | 26 | 17 | 10 |
| **8** | 3957 | 43 | 40 | 7 | 10 |
| **9** | 3926 | 45 | 31 | 6 | 18 |
| **10** | 0 | 35 | 33 | 13 | 19 |
| **11** | 1685 | 63 | 24 | 8 | 5 |
| **12** | 602 | 52 | 30 | 14 | 4 |
| **13** | 416 | 38 | 30 | 14 | 18 |
| **14** | 3805 | 32 | 27 | 24 | 17 |
| **15** | 3625 | 57 | 28 | 10 | 5 |
| **16** | 1136 | 36 | 48 | 8 | 8 |
| **17** | 577 | 36 | 44 | 6 | 14 |
| **18** | 0 | 33 | 17 | 39 | 11 |
| **19** | 518 | 14 | 26 | 46 | 14 |
| **20** | 0 | 37 | 35 | 12 | 16 |
| **21** | 798 | 43 | 38 | 10 | 9 |
| **22** | 1715 | 38 | 33 | 15 | 14 |
| **23** | 610 | 62 | 18 | 14 | 6 |
| **24** | 193 | 36 | 27 | 20 | 17 |
| **25** | 801 | 45 | 39 | 11 | 5 |
| **26** | 994 | 38 | 33 | 17 | 12 |
| **27** | 1805 | 47 | 29 | 16 | 8 |
| **28** | 1394 | 29 | 47 | 10 | 14 |
| **29** | 710 | 53 | 25 | 11 | 11 |
| **30** | 0 | 49 | 27 | 10 | 14 |
| **31** | 1140 | 36 | 30 | 20 | 14 |
| **32** | 0 | 58 | 24 | 6 | 12 |
| **33** | 1127 | 31 | 38 | 18 | 13 |

**Supplementary Table 6. Air borne solvents concentrations in workshops**

| **Car paint shop** | **N°** | **Air borne solvents concentrations** | | |
| --- | --- | --- | --- | --- |
|  |  | **Benzene** mg/L | **Toluene** mg/L | **Xylene** mg/L |
| 10 | **1** | Not sampled | Not sampled | Not sampled |
|  | **2** |  |  |  |
|  | **3** |  |  |  |
|  | **4** |  |  |  |
|  | **5** |  |  |  |
|  | **6** |  |  |  |
| 1 | **7** | 0,330 | 10,74 | 28,92 |
|  | **8** | 0,330 | 10,74 | 28,92 |
|  | **9** | 0,330 | 10,74 | 28,92 |
|  | **13** | 0,330 | 10,74 | 28,92 |
|  | **14** | 0,330 | 10,74 | 28,92 |
| 2 | **10** | 0,310 | 27,22 | 115,20 |
|  | **11** | 0,310 | 27,22 | 115,20 |
|  | **12** | 0,310 | 27,22 | 115,20 |
| 3 | **15** | 0,180 | 9,55 | 19,34 |
| 4 | **16** | 0,300 | 25,27 | 101,54 |
|  | **17** | 0,300 | 25,27 | 101,54 |
|  | **18** | 0,300 | 25,27 | 101,54 |
|  | **19** | 0,300 | 25,27 | 101,54 |
| 5 | **20** | 0,100 | 13,20 | 52,60 |
| 6 | **21** | 0,430 | 21,50 | 108,35 |
| 7 | **22** | 0,340 | 8,25 | 70,12 |
|  | **23** | 0,340 | 8,25 | 70,12 |
|  | **24** | 0,340 | 8,25 | 70,12 |
| 8 | **25** | 0,200 | 12,80 | 35,70 |
|  | **26** | 0,200 | 12,80 | 35,70 |
| 9 | **27** | 0,100 | 14,50 | 67,30 |
|  | **28** | 0,100 | 14,50 | 67,30 |
|  | **29** | 0,100 | 14,50 | 67,30 |
|  | **30** | 0,100 | 14,50 | 67,30 |
|  | **31** | 0,100 | 14,50 | 67,30 |
|  | **32** | 0,100 | 14,50 | 67,30 |
|  | **33** | 0,100 | 14,50 | 67,30 |

**Supplementary Table 7. Exposed cohort. Comet score data**

| N° | Comet Length (µm) | Comet Height (µm) | Comet Area (µm) | Head Diameter (µm) | Head Area (µm) | %DNA in Head | Tail Length (µm) | Tail Area (µm) | %DNA in Tail | Tail Moment |
| --- | --- | --- | --- | --- | --- | --- | --- | --- | --- | --- |
| 1 | 170,8 | 106 | 10590 | 65,4 | 3653 | 37,4 | 105,4 | 6936 | 62,6 | 82,7 |
| 2 | 168,2 | 120,4 | 12487 | 50,2 | 4046 | 47,4 | 118 | 8440 | 52,6 | 89,4 |
| 3 | 139,8 | 79,1 | 7958 | 58,4 | 4018 | 65,8 | 81,4 | 3940 | 34,2 | 77,1 |
| 4 | 218,1 | 107,1 | 15773 | 68,6 | 4264 | 42 | 149,5 | 11508 | 58 | 122,5 |
| 5 | 152,2 | 75 | 8271 | 67 | 4413 | 66,1 | 85,2 | 3858 | 33,9 | 62,5 |
| 6 | 189,7 | 112,3 | 15557 | 50,8 | 3669 | 25,6 | 138,9 | 11887 | 74,4 | 112,7 |
| 7 | 94 | 75 | 4042 | 37,2 | 1718 | 39,2 | 56,8 | 2324 | 60,8 | 36 |
| 8 | 147,2 | 83,5 | 4520 | 46,8 | 1728 | 42,4 | 100,4 | 2791 | 57,5 | 67,5 |
| 9 | 188,1 | 89,3 | 8481 | 63,6 | 3337 | 51 | 124,5 | 5144 | 49 | 72,8 |
| 10 | 156,3 | 85,3 | 7013 | 60,2 | 3293 | 50,4 | 93,4 | 3720 | 49,6 | 50,2 |
| 11 | 118,3 | 91,2 | 7765 | 79,1 | 5374 | 73,6 | 37,3 | 2391 | 26,4 | 10,54 |
| 12 | 134 | 80 | 5863 | 72,4 | 4528 | 80,8 | 61,5 | 1334 | 21,3 | 22,54 |
| 13 | 127,3 | 85 | 6633 | 68,4 | 4088 | 63,6 | 59 | 2545 | 36,4 | 22,33 |
| 14 | 91,3 | 87,1 | 5836 | 80 | 5289 | 92 | 11,3 | 546 | 8,7 | 2,5 |
| 15 | 107,6 | 77,6 | 5284 | 48,2 | 2786 | 50,4 | 59,4 | 2498 | 49,5 | 35,3 |
| 16 | 192,4 | 92,9 | 7442 | 42,7 | 1844 | 25,2 | 149,7 | 5598 | 77,3 | 118 |
| 17 | 137,3 | 85,3 | 9636 | 57,6 | 3909 | 51,5 | 79,7 | 5727 | 48,5 | 49,3 |
| 18 | 220,7 | 76,3 | 4249 | 47,5 | 2424 | 52,9 | 173,2 | 2067 | 57,6 | 162,2 |
| 19 | 178 | 93,4 | 7135 | 42,9 | 1945 | 30 | 135,1 | 5190 | 73 | 107 |
| 20 | 163,5 | 87,9 | 8583 | 54,4 | 3694 | 49,6 | 109,1 | 4889 | 50,4 | 70,65 |
| 21 | 146 | 87 | 6316 | 45 | 2116 | 42 | 100,7 | 4210 | 62,1 | 75,6 |
| 22 | 96 | 78 | 7125 | 76 | 6724 | 90,4 | 20 | 400 | 9,6 | 3,2 |
| 23 | 97 | 74 | 6798 | 75 | 6567 | 93,6 | 23,7 | 241 | 7 | 4 |
| 24 | 78 | 66 | 3527 | 67 | 3124 | 90 | 13,9 | 403 | 16,8 | 5,8 |
| 25 | 80 | 65 | 4687 | 60 | 4179 | 85,3 | 20 | 508 | 14,7 | 3,7 |
| 26 | 112 | 66 | 4240 | 55,6 | 3077 | 64,7 | 56 | 1163 | 35,3 | 33 |
| 27 | 160 | 126 | 4846 | 60 | 2246 | 44,5 | 100,4 | 2600 | 55,5 | 59 |
| 28 | 203 | 71 | 4802 | 49,5 | 2621 | 60,6 | 153,4 | 2443 | 51,4 | 151 |
| 29 | 123 | 72 | 4033 | 55,6 | 2350 | 62,5 | 67,6 | 1683 | 37,5 | 58,7 |
| 30 | 148 | 96 | 7144 | 58 | 2479 | 48,2 | 90 | 4665 | 51,7 | 77,8 |
| 31 | 162 | 82 | 7176 | 61,2 | 3125 | 55,8 | 101 | 4051 | 49,7 | 57,2 |
| 32 | 174 | 79 | 6888 | 63,6 | 2781 | 53,5 | 110 | 4107 | 51,8 | 67,3 |
| 33 | 136 | 68 | 3792 | 48,6 | 1560 | 53 | 87,2 | 2232 | 52,2 | 51,8 |

**Supplementary Table 8. Non-exposed cohort. Comet score data**

| N° | Comet Length (µm) | Comet Height (µm) | Comet Area (µm) | Head Diameter (µm) | Head Area (µm) | %DNA in Head | Tail Length (µm) | Tail Area (µm) | %DNA in Tail | Tail Moment |
| --- | --- | --- | --- | --- | --- | --- | --- | --- | --- | --- |
| 1 | 73 | 59 | 3386 | 50 | 1946 | 83 | 26 | 1439 | 22 | 8 |
| 2 | 49,6 | 48 | 1651 | 45,1 | 1519 | 90,4 | 7,5 | 132 | 15,8 | 3,8 |
| 3 | 49 | 48 | 1676 | 46 | 1568 | 93 | 4 | 109 | 12 | 1 |
| 4 | 139 | 82 | 9631 | 54 | 3385 | 56 | 84 | 6246 | 44 | 71 |
| 5 | 73,1 | 60 | 3704 | 51,6 | 2438 | 84,5 | 21,5 | 1275 | 15,5 | 19 |
| 6 | 76,3 | 61,8 | 3225 | 59,1 | 2589 | 88,5 | 21,5 | 636 | 17,8 | 19,7 |
| 7 | 46,2 | 45 | 1302 | 42,8 | 1193 | 92,1 | 4,25 | 109 | 9,7 | 4 |
| 8 | 47,3 | 43,5 | 1365 | 42 | 1241 | 91,6 | 7,6 | 124 | 10,5 | 2,4 |
| 9 | 60 | 58,7 | 2586 | 56,4 | 2484 | 97,2 | 4 | 102 | 5,6 | 6,7 |
| 10 | 80,6 | 60,3 | 2876 | 56,8 | 2396 | 89,4 | 23,8 | 480 | 17,1 | 10,3 |
| 11 | 95 | 69 | 5977 | 53,2 | 3025 | 76 | 52,1 | 2953 | 30 | 31 |
| 12 | 55,2 | 56 | 2088 | 53,3 | 2034 | 97 | 2,4 | 53 | 6,46 | 5,9 |
| 13 | 59,3 | 55,7 | 2197 | 50,6 | 1883 | 86,7 | 8,7 | 313 | 14,6 | 2,8 |
| 14 | 76,3 | 62 | 2930 | 56,6 | 2394 | 85,5 | 19,7 | 537 | 14,5 | 5,5 |
| 15 | 65 | 53 | 1990 | 51 | 1726 | 92,2 | 14,2 | 264 | 8 | 12,3 |
| 16 | 87 | 63,5 | 3471 | 57,6 | 2547 | 80 | 29,4 | 924 | 20 | 18,7 |
| 17 | 73 | 68 | 3577 | 62,6 | 3165 | 88,5 | 12,8 | 412 | 16,3 | 4,2 |
| 18 | 111 | 70,4 | 4351 | 57,4 | 2651 | 65 | 53,7 | 1736 | 35 | 31,8 |
| 19 | 154 | 89 | 7360 | 45,5 | 2400 | 39 | 109 | 4960 | 65 | 81,3 |
| 20 | 60 | 60 | 2678 | 55,4 | 2497 | 95 | 4,6 | 181 | 5,6 | 1,25 |
| 21 | 58,4 | 55,7 | 2170 | 53,6 | 2021 | 93 | 5,3 | 149 | 7,7 | 1,4 |
| 22 | 114,4 | 71,3 | 5406 | 68,7 | 4057 | 80,3 | 45,6 | 1349 | 23,6 | 41,3 |
| 23 | 97 | 56,5 | 4188 | 58,4 | 2983 | 80,6 | 38,5 | 1204 | 19,4 | 17,3 |
| 24 | 109 | 57,3 | 3062 | 50,6 | 2224 | 73,4 | 58,3 | 838 | 26,6 | 32,1 |
| 25 | 79,1 | 59,1 | 2958 | 54,2 | 2421 | 86,2 | 25 | 537 | 15,5 | 7 |
| 26 | 88,7 | 60,7 | 3328 | 57,3 | 2376 | 76,5 | 31,4 | 953 | 23,5 | 18 |
| 27 | 59,4 | 56 | 2574 | 55,1 | 2454 | 96,4 | 4,3 | 120 | 5 | 2 |
| 28 | 128,4 | 71 | 6881 | 58 | 3703 | 66,4 | 70,4 | 3177 | 33,6 | 35,9 |
| 29 | 136,2 | 73,3 | 4969 | 66,6 | 3622 | 75,3 | 69,5 | 1347 | 26 | 7,6 |
| 30 | 87 | 59,5 | 3342 | 59,5 | 2556 | 84,7 | 27,1 | 786 | 15,3 | 14,9 |
| 31 | 93,3 | 62,1 | 3764 | 54,2 | 2514 | 76,7 | 39,1 | 1250 | 23,3 | 21 |
| 32 | 96 | 62,7 | 4202 | 57 | 2797 | 76,9 | 38,5 | 1406 | 23,1 | 18,5 |
